# Health outcomes, education, healthcare delivery and quality – 3056. Constructed supporting program improves asthma treatment outcomes in children

**DOI:** 10.1186/1939-4551-6-S1-P224

**Published:** 2013-04-23

**Authors:** Jing-Long Huang, Shu-Hsien Chen, Kuo-Wei Yeh

**Affiliations:** 1 Department of Pediatrics, Chang Gung Memoiral Hospital, Taipei, Taiwan; 2 Department of Nursing,, Chang Gung Memorial Hospital At Keelung, Keelung, Taiwan; 3 Division of Pediatric Allergy Asthma and Rheumatology, Department of Pediatrics, Chang Gung Memorial Hospital, Taoyuan, Taiwan

## Background

Children with asthma and their parents usually can not cope with this disease leading to poor control. A constructed supporting program can improve treatment outcomes.

## Methods

We conducted an open-label randomized controlled trial to investigate the impact of support intervention to the outcome of parents of asthmatic children. The constructed supporting program includes scheduled one-by-one and group asthma knowledge education, treatment adherence reinforcement, family support course, and telephone follow-up. The differences of parental knowledge of asthma, medication adherence, hospital re-admission, and health care resource usage between two groups were compared.

## Results

The study enrolled 130 parents of asthmatic children who were randomized into 2 groups in the Pediatric Allergic Clinic of the Chang Gung Memorial Hospital. The experimental group (65 parents) who received support program and training course in addition to the regular care provided to the control group. There was less emergency room visits in experimental group (6 /month, p<0.05). The understanding of the disease was much improved in parents of experimental group (16.09±1.04 versus 11.91±2.14, p<0.01). Furthermore, parents acquired a more positive attitude to asthma and had better adherence The control group without supporting program presented with irregular follow-up and poor compliance of medication usage.

## Conclusions

This study emphasizes that a support program in children with asthma must be an important part of treatment and it can reduce unpredicted heath care resource usage. used to educate parents in how to provide the best treatment plan for their children. It may play a significant role in reducing asthma progression and morbidity of late stage asthmatic children.

